# G-quadruplexes: a promising target for cancer therapy

**DOI:** 10.1186/s12943-021-01328-4

**Published:** 2021-02-25

**Authors:** Nils Kosiol, Stefan Juranek, Peter Brossart, Annkristin Heine, Katrin Paeschke

**Affiliations:** Department of Oncology, Hematology, Rheumatology and Immune-Oncology, University Hospital Bonn, 53127 Bonn, Germany

**Keywords:** G-quadruplex, genome instability, cancer progression, cancer therapy, malignant melanoma, pancreatic cancer, acute myeloid leukemia

## Abstract

DNA and RNA can fold into a variety of alternative conformations. In recent years, a particular nucleic acid structure was discussed to play a role in malignant transformation and cancer development. This structure is called a G-quadruplex (G4). G4 structure formation can drive genome instability by creating mutations, deletions and stimulating recombination events. The importance of G4 structures in the characterization of malignant cells was currently demonstrated in breast cancer samples. In this analysis a correlation between G4 structure formation and an increased intratumor heterogeneity was identified. This suggests that G4 structures might allow breast cancer stratification and supports the identification of new personalized treatment options. Because of the stability of G4 structures and their presence within most human oncogenic promoters and at telomeres, G4 structures are currently tested as a therapeutic target to downregulate transcription or to block telomere elongation in cancer cells. To date, different chemical molecules (G4 ligands) have been developed that aim to target G4 structures. In this review we discuss and compare G4 function and relevance for therapeutic approaches and their impact on cancer development for three cancer entities, which differ significantly in their amount and type of mutations: pancreatic cancer, leukemia and malignant melanoma. G4 structures might present a promising new strategy to individually target tumor cells and could support personalized treatment approaches in the future.

## Introduction

Cancer is the world’s second most leading cause of death [1]. Although therapeutic strategies for many cancers have greatly advanced during the last years, still about 9.6 million people died of cancer in 2018 [2]. This highlights the need to strengthen the research on causes of cancer development and to improve diagnostic and anti-tumor treatment options. Different promising therapeutic strategies have been identified in the last decades. Among those, immunotherapy and targeted therapies have revolutionized anti-tumor therapy [3]. The identification of genetic and epigenetic abnormalities as well as tumor-growth promoting oncogenes in tumor cells provided the rationale for molecular targeted therapies [4, 5]. Immune-oncology (IO) aims at boosting the patient´s own immune system to eliminate tumor cells. One example is the immune checkpoint blockade [6]. Both approaches, targeted therapies as well as IO, significantly improved survival outcome in cancer patients, such as in malignant melanoma and other cancer entities [7, 8]. A diverging new strategy aims to modulate the 3-dimensional structure of the DNA with the goal to influence biological processes and genome stability.

Genomic DNA canonically adopts a standard B-DNA conformation. DNA can also fold into alternative structures such as DNA hairpins, holiday junctions, cruciforms, triplexes or G-quadruplexes (G4) [9, 10]. Although the relevance of G4 structures in living cells was controversially discussed in the past, accumulating experimental data now supports the existence and importance of these structures in living cells [10–12].

G4 structures can form within DNA and RNA [13]. In a G4 structure, four guanines are held together by Hoogsteen hydrogen bonds wherein each guanine can act as a donor and acceptor for two hydrogen bonds. Based on *in vitro* experiments it was predicted that G4 structures form in regions harboring a specific G4 motif: G_≥3_N_1-7_G_≥3_N_1-7_G_≥3_N_1-7_G_≥3_ [13]. However, current experimental data shows that G4 structures can also form within regions that have longer loops or less than 3 guanines per repeat as well as in regions that do not follow this stringent G4 motif [14, 15]. Among different factors the stability of the G4 structure depends on the numbers of guanines per repeat and the length of the loops [13, 16].

Computational as well as deep-sequencing approaches have demonstrated that in the human genome over 700.000 regions exist that could potentially fold into G4 structures [14, 15, 17, 18]. Also in other organisms including viruses, yeasts and different bacterial genomes regions with a strong potential to fold into G4 were mapped genome-wide [15, 19–21]. To this date, the identification of a G4 motif within the genome does not proof the formation of G4 structures at these regions *in vivo*, but simply gives a potential to form a G4. G4 formation needs to be experimentally evaluated and depends on different factors (e.g. protein binding, loss of protein binding, cell cycle phase, stress), of which not all are known, yet.

**Fig. 1:**
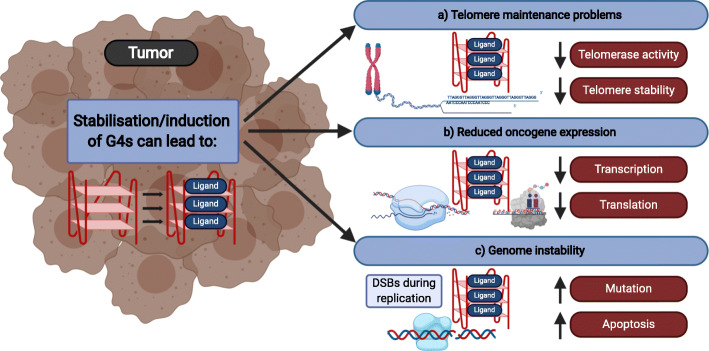
Schematic overview of the effects of G4 ligands on cancer cells. Most G4 ligands cause slow growth. These growth changes are the consequence of alteration within biological processes. Depending on the ligand and cell type G4 stabilization can lead to changes in **a** telomere maintenance **b** gene expression of oncogenes **c** increased genome instability. Created with BioRender.

G4 motifs are not randomly distributed throughout the genome, but are enriched in certain regions (e.g. promoters, telomeres, transcription factor binding sites) [14, 22, 23]. More than 40% of human promotor regions harbor at least one G4 motif [24]. The evolutionary conservation, the specific location within the genome [15, 19, 25] as well as different biochemical and molecular experiments underline the current model that G4 structures form in living cells, where they support/affect different biological pathways (e.g. protein expression, telomerase activity and genome stability) (Fig. 1) [10–12].

The physiological relevance of G4 structures is further supported by the existence of proteins that are able to bind and unfold G4 structures [11, 12, 26]. There are three classes of G4-interacting proteins described in the literature: G4 binding, G4 stabilizing and G4 unwinding proteins (e.g. helicases: BLM, WRN, BRIP1/FANCJ and PIF1) [27]. It has been reported that mutations and/or deletions of these proteins (e.g. PIF1) lead to changes in the formation of G4 structures. This in turn can result in changes of biological pathways (transcriptional changes) and can also increase genome instability [28–32]. This agrees with the finding that changes within some G4-interacting helicases are linked to cancer progression and tumorigenesis [27]. However, the link of tumorigenesis and mutations of helicases is not proofed to be related to G4 formation. Antibody stainings as well as G4 ChIP-seq in stomach and liver tissues of immortalized HaCaT cells showed increased levels of G4 structures compared to a normal/healthy state [18, 33, 34]. Similar observations were made for other cancer states. Different lines of evidence demonstrated that changes in G4 formation/stability can alter telomerase activity [35, 36], transcription efficiency (inhibit or promote [11, 37, 38]), stall DNA replication and induce genome instability [39, 40]. Changes can be triggered chemically (G4-ligands) or by proteins that modulate G4 formation. G4 ligands that modulate G4 structure formation or stabilize G4 structures were developed with the idea that G4 formation can be used as an anti-tumor treatment strategy by blocking cellular replication or expression of oncogenes [41, 42]. To date, about 1000 different G4 ligands have been identified [43]. These ligands differ in their specificity, binding surface and cell permeability [44]. Most of them, including MM41 [45], Telomestatin [46], BRACO19 [47], TMPyP4 [48], RHPS4 [49] and PDS [50] preferentially bind G4 structures over duplex DNA. However, most of these binding preferences were tested *in vitro* and it is difficult to elucidate if and how selective these G4 ligands perform in living cells. Additionally, different lines of evidence indicate that at least some G4 ligands (e.g. BRACO19) also bind to other non-canonical DNA structures such as the i-motif [51, 52], indicating the possibility that some of the G4 ligand-mediated effects in living cells might not only be caused by G4 stabilization. Regardless, some ligands show very promising results as novel therapeutic drugs [41, 42], but their use in clinical applications is not approved, yet. This is mainly due to selectivity problems and the fact that most G4 ligands target multiple G4s and by this effect many different sites in the genome. In the last years, tremendous effort has taken place to investigate G4 ligands that selectively target specific G4 structures with the aim that these ligands have high anti-tumor activity but reduced side effects [53, 54].

### G-quadruplex structures and cancer

In the last years, formation and/or stabilization of G4 structures has been discussed as a potential therapeutic tool against tumor cells [41, 42, 55]. In most of these reports, G4 formation/stabilization was supported by G4 ligands. Three main therapeutic strategies have been investigated so far (Fig. 1). First, G4 formation/stabilization at telomeres was used as a tool to block telomerase activity (Fig. 1a). In about 85-90% of all cancer cells telomerase activity is upregulated, which allows the cell to replicate without telomere shortening [56]. G4 structures at telomeres alter telomerase binding and block telomerase activity *in vitro* [57] and *in vivo* [58–60]. The working hypothesis is that G4 formation at telomeres can be used to block telomerase in tumors and by this prevent uncontrolled DNA replication, while somatic cells do not express telomerase and are thus not affected. Until now G4 stabilization at telomeres has been studied by using different ligands [35]. Most of them cause reduced growth of targeted tumor cells by influencing different telomere maintenance factors [35, 61]. For example, treatment of cancer cells with the G4 ligands Telomestatin [62] or 2,6-diamidoanthraquinone derivatives [63] leads to telomerase inhibition, whereas the G4 ligand RHPS4 resulted in telomere dysfunction by disrupting the telomere protecting shelterin complex [64]. These uncapped telomeres are repaired in dependency of PARP1. Co-treatment of cells with RHPS4 and the PARP1 inhibitor GPI-15427 increased the growth reduction observed upon RHPS4 treatment, indicating the biomedical significance of this finding [65]. A novel approach is to use specific G4 ligands that act as photosensitizer to facilitate photodynamic therapy (PDT) [66]. Photosensitizer aim to specifically target tumors and cause an increased ROS production after photo-irradiation, which results in a cytotoxic effect for the tumor cells. Porphyrins are well known photosensitizer used in PDT. Specific porphyrin derivates have been developed to target telomeric G4 (TMPipEOPP [67], ZnP1 [68]). TMPipEOPP binds to telomeric G4. After photoinduction, TMPipEOPP-bound sites are cleaved, which leads to increased ROS levels and cell death [67]. ZnP1 acts via a different mechanism and creates a singlet oxygen after photoinduction that drives cleavage, ROS production and cell death [68].

Second, G4 formation was also discussed as a supporting element that impacts gene expression of oncogenes [38, 53, 69, 70] (Fig. 1b). This hypothesis was underlined by the observation that most promoters of oncogenes harbor more G4 motifs than promoters of regulatory or tumor suppressor genes [70, 71]. *In vitro* and *in cellulo* experiments revealed that changes within G4 structure formation in promoters correlates with a reduction in gene expression (e.g. *MYC* [72], *VEGF* [73], *BCL2* [74], *KRAS* [75] and *KIT* [72, 76, 77]). Especially the G4-mediated changes in *MYC* expression [72, 76] have been studied extensively. The *MYC* gene encodes for the transcription factor MYC, which is upregulated in 70% of all cancers [78] and drives oncogenesis by altering cell proliferation, metabolism, and immune evasion [78–80]. Due to the lack of direct inhibitors, current strategies aim to regulate the gene expression of *MYC* itself [81]. One attempt is to block its expression by inducing G4 structures that are located within the *MYC* gene promoter [72, 76, 82]. Different G4 ligands have been tested to regulate *MYC* expression. Global studies using these G4 ligands resulted in reduced tumor growth, which was correlated to decreased expression of *MYC* and other oncogenes [54, 83]. But many of these ligands are not selective and it is not clear if the G4 ligand-dependent mechanism is via a MYC-dependent or another, yet unknown mechanism [84].

Third, under specific conditions G4 structures can cause genome instability [10, 11] (Fig. 1c). In detail, mis-regulated G4 structures, which form at the “wrong” time and place in the cell, cause alterations within DNA replication and can induce DNA damage and recombination events [39, 40, 85]. These findings agree with observations that treatment with G4 ligands (e.g. PDS) leads to enhanced DNA double strand breaks, replication pauses, micronuclei formation and telomere maintenance problems [30, 50, 65, 86, 87]. The enhanced mutagenic rate, which is stimulated by G4 structures, is favored in cells that lack functional G4-unwinding helicases (e.g. FANCJ, BLM) [28, 88–91]. This evidence raised the idea that genetic alterations (e.g. point mutations, insertion, deletion, recombination events, telomere addition or even epigenetic changes), which are observed in many cancers, might be stimulated by G4 formation. If this assumption is correct, therapeutic strategies using G4 ligands will cause increased genome instability.

Increased genome instability has a dual role in cancer research. On the one hand it stimulates tumorigenesis, but on the other hand genome instability is used as a therapeutic approach to induce apoptosis and autophagy in tumor cells (e.g. radiation therapy). A number of publications demonstrated that treatment with G4 ligands is correlated with enhanced DNA damage, telomere dysfunction and DNA damage checkpoint activation by ATM [30, 50, 92–94]. Treatment of cells with the G4 ligand 20A led to a G4-mediated upregulation of genes associated with apoptosis and autophagy, which underlines the anti-tumorigenic effect of 20A [93]. Stabilization of G4 structures by PDS in cells that were deficient in homologous recombination (HR) (e.g. BRCA1, BRCA2, Rad51 deficient cells) exhibited slow growth, fragile telomeres, DNA double strand breaks and checkpoint activation [94]. The authors concluded that in the absence of a functional DNA repair machinery G4 stabilization by PDS exacerbates genome instability, which further drives checkpoint activation, G2/M cell-cycle arrest and cell death [86]. This data underlines the hypothesis that G4 stabilization might be a promising tool to target HR-deficient tumors, even for those that are resistant to PARP inhibition (olaparib) [94]. At this point the G4 ligand CX-5461 [95, 96] is in phase I clinical trials for patients with BRCA1/2 deficient tumors, which is deficient for HR. CX-5461 acts as a topoisomerase II inhibitor that impacts DNA break production [97]. Also, other G4 ligands have been tested in combination with DNA damaging therapies. Co-treatment of PDS with the PRKDC inhibitor NU7441 resulted in enhanced growth defects. PRKDC (also known as DNA-PK) is crucial for non-homologous end joining (NHEJ) [98].

ATRX-deficient glioma cells accumulate replication problems and DNA damage due to the lack of the helicase ATRX [90, 99–101]. Most likely the DNA damage is favored by G4 structures [102, 103]. Treatment with the G4 ligands PDS, CX-5461 or CX-3543 leads to even more elevated DNA damage. In combination with DNA damaging agents (IR or hydroxyurea) the cytotoxic effect becomes further accelerated [90]. The question is how and if G4 structure-mediated DNA damage might be a potential tool to modulate DNA damage as a treatment option.

### G-quadruplexes - a tool for anti-cancer therapy prospects?

The current knowledge of G4 DNA suggests that the anti-tumor effects of different G4 ligands relies on changes within telomere maintenance (Fig. 1a) (telomere damage or telomerase regulation), changes of oncogene expression (Fig. 1b) and increased genome instability (Fig. 1c). While functional telomeres and active oncogene expression is known to be important for the development of malignancies, DNA-damaging agents are usually considered cancerogenic. Genome instability and mutations are considered one of the ten hallmarks of cancer [104]. Different cancer types vary in the amount of acquired somatic mutations [105]. In consequence, some cancers rely on alterations in a small and defined subset of cellular pathways, while other cancers, such as melanoma, are very versatile. This is considered an important explanation for the development of resistance to targeted therapies and immunotherapies and highlights three important points. First, G4 stabilization drives genome instability and the cytotoxic effect of G4 stabilization is enhanced in cells that accumulate DNA damage (e.g. due to IR treatment or the loss of a functional repair pathway – see above). This raises the question whether the mutational burden of a tumor has an impact on the outcome of G4 ligand treatment. Second, what long-term effects does the treatment with G4 ligands have on tumor development? Third, does the G4 landscape change during therapy and does this contribute to genome instability that supports tumor relapse?

To this date little is known regarding the risks of long-term treatment and how G4 formation is changed over time during treatment and how/if this contributes to tumor relapse. We believe that these questions are of high relevance and that they will be addressed in the near future. Here, we aim to investigate if the current literature allows us to address these questions. We chose three different cancer entities that vary in their mutational burden and have been used to study G4 structure-mediated changes of cancer growth.

### G-quadruplexes and melanoma

Melanoma is the most aggressive skin cancer. While the 5-year overall survival (OS) rate of localized melanoma is about 99%, it decreases for distant metastatic melanomas to only about 25% [106]. The development of targeted therapies [107, 108] and new immunotherapeutic approaches [109] have significantly improved survival rates, but most patients still die [110].

Melanomas are generally characterized by a high mutational burden [111, 112] and have frequently been used as an example for a highly mutated cancer that blocks DNA-damage induced apoptosis by mutations in different proteins, including TP53 [113, 114], POU3F2/BRN2 [115], RHOJ [116] and RSK [117]. Despite this, some prevalent mutated genes such as *BRAF* (50% of non-chronic sun damage melanomas) [118, 119] and *NRAS* (20-30%) [110, 120] have been identified. *NRAS* is also known to harbor a potential G4 motif [121].

Many studies have used different melanoma cell lines to test the relevance and effect of G4 structure stabilization on melanoma cell growth. As shown in Table 1, a variety of studies were conducted using different G4 ligands in mouse and human melanoma cell lines.

**Table 1:** Overview of studies investigating the effect of G4 ligands on different melanoma cell lines

Ligand/G4 targeting	Cell line (X_**h**_=human, X_**m**_=mouse)	Treatment duration (***in vitro***)	Growth Effect	Cellular Effects				Literature
				Telomere maintenance	Oncogene regulation	Genome instability	Not tested	
5ME	B16F10_m_	48 h	IC_50_: ~100 μM			x		[122]
Ant1,5	SKMel-5_h_, ROSS_h_, A375_h_, M14_h_	48 h	IC_50_: 4 μM (ROSS) to 10 μM (SCMel-5)	x				[123]
BRACO19	B16_m_	24 h	IC_50_: 28 μM				x	[124]
C8	B16_m_	72 h	IC_50_: ~5 μM				x	[125]
C14 Derivate from TMPyP4; induces singlet oxygen (^1^O_2_) production	B78-H1_m_	n.a.	*In vitro*: IC_50_ (metabolic activity): 10 nM *In vivo*: About 50% tumor growth reduction.		x			[121]
c-exNDIs	A375_h_, SK-MEL-2_h_	48 h	IC_50_: ~8 nM		x			[126]
CORON	M14h, LOX IMVI_h_, MALME-3M_h_, SK-MEL-2_h_, SK-MEL-28_h_, SK-MEL-5_h_, UACC-62_h_	n.a.	LC_50_: mean for all cell lines ~2.5 μM	x	x	x		[127, 128]
CX-3543 (Quarfloxin)	M14_h_, MALME-3M_h_, UACC-257_h_, UACC-62_h_	n.a.	IC_50_: **>** 1 μM for all cell lines		x	x		[129]
EMICORON	M14_h_, LOX IMVI_h_, MDA-MB-435_h_, SK-MEL-2_h_, SK-MEL-28_h_, SK-MEL-5_h_, UACC-62_h_, UACC-257_h_	n.a.	LC_50_: mean for all cell lines ~3 μM	x	x	x		[127, 128]
IZCZ-0	A375_h_	24 h	IC_50_: 2.3 μM		x			[130]
IZCZ-3	A375_h_	24 h	IC_50_: 4.2 μM		x			[130]
IZTC-1	B16_m_	24 h	IC_50_: ~2.2 μM, ~50-65% reduced melanoma growth *in vivo.* Binds preferentially to MYC G4		x			[124]
N,N'-bis(3,4-dihydroxbenzy lidene)-1,2-diaminobenzene (crosslinker)	B16F1_m_	n.a.			x			[131]
Naphthalene diimide derivatives (compound 2)	SKMEL-5_h_	48 h	IC_50_: 1.7 μM	x		x		[132]
PhenDC3	A375_h_	96 h	GI_20_: 10 μM				x	[69]
Phenyl 1,2,3-triazole-thymidine ligands (L1, L2, L3)	B16F10_m_	24 and 48 h	IC_50_: 200 μM (L1), 125 μM (L2), 50 μM (L3)	x				[133]
PPL3C	M14_h_	96 h	IC_50_: 0.8 μM	x				[128, 134]
Pyridostatin	A375_h_	96 h	GI_20_: 1.5 μM				x	[69]
RHPS4	M14_h_, PLF2_h_, JR1_h_, JR8_h_, SBCL1_h_, SAN_h_, LP_h_, LM_h_, JR5_h_, M14_h_	5 and 7 days	IC_50_: 3.1 μM (5 days M14), ~1 μM (5 days PLF2_h_, JR1, JR8, SBCL1, SAN) ~1 μM (7 days M14, PLF2_h_, JR1, JR8, SBCL1, SAN) About 50% reduced tumor growth in melanoma xenografts	x				[135–137]
TMPyP4	B78-H1_m_	48 h	IC_50_ (metabolic activity): 200 nM IC_50_: 85 μM ~65% reduced tumor growth *in vivo* (+ light therapy)		x			[121, 122]
trans-resveratrol (tRES)	M14_h_, SKMEL-28_h_	48 and 72 h	IC_50_: 5 μM (M14, 48 h), 2.5 μM (SKMEL-28, 48 h) IC_50_: ~25 μM (SKMEL-28, 72 h)	x	x			[138, 139]
Trisubstituted naphthalimides (compounds VII, VIII and IX)	M14_h_	5 days	IC_50_: ~1.5 μM (VII), ~34.7 μM (IX)	x				[140]

Treatment with the G4 ligand RHPS4 leads to the inhibition of cancer cell growth in several different melanoma cell lines [135]. Also, treatment with the imidazole-benzothiazole conjugate IZTZ-1 reduces the growth of melanoma cells [124]. Both ligands target *MYC* and lead to a reduction of MYC protein expression (up to 80%) after G4 stabilization [124, 131]. A systematic screen of different naphthalene diimides revealed that “G4 ligand 1” targets the G4s within *KIT* and *BCL2* and drives melanoma tumors into a pro-apoptotic environment. In this paper the authors propose that G4 stabilization might be relevant in the context of resistance to targeted therapies of *BRAF* mutant melanomas [126]. However, if the ligands target other promoters and stimulate changes in other pathways (e.g. telomeres) has not been investigated in this context, yet. A similar growth effect was observed after treatment of B78-H1-tumor-bearing mice with the G4 ligands C14 and TMPyP4 [121]. Both ligands are photosensitizers that caused retarded tumor growth and increased survival time of irradiated mice. Based on their data, the authors propose a model in which photocleavage of G4 RNA at the mitogenic *Ras* gene was stimulated due to TMPyP4 and C14 binding, which led to reduced levels of RAS protein [141, 142]. Overall these studies measured enhanced apoptosis and necrosis [121]. It is not clear whether the observed effects were only because of changes within the mRNA of the *Ras* genes or if additional targets such as *Myc* and other genes also drove this effect.

Note, treatment with either RHPS4 or BRACO19 for 3-21 days led to telomere uncapping and end-to-end fusion of chromosomal ends in both, prostate and melanoma cell lines [65]. In contrast, in a UXF1138L uterus carcinoma cell line telomere uncapping could already be detected after 24 h of treatment with a G4 ligand [64]. The authors speculate that this difference in treatment timing might come from the difference in telomere length between prostate/melanoma cells (4-10 kb) and uterus carcinoma cells (2.7 kb). This raises the hypothesis that telomere length as well as telomerase activity might be useful markers for the success of G4 stabilization by ligands [64].

In an alternative approach guanine-rich oligos (t-oligos) homolog to the telomeric overhang that forms the G4 structures were designed [143]. Within melanoma cells the t-oligos led to a decreased proliferation rate, enhanced apoptosis and reduced expression of the catalytic subunit of the telomerase reverse transcriptase (TERT). Two major telomere-binding proteins, POT1 and TERF2, bind to these t-oligos and might cause the observed effects (107). Another study investigated the effect of TERF2 on cancer proliferation and genome stability [136]. They observed that TERF2 inhibition reduces tumor growth in dependency to its telomere capping potential. This implies that cells with shorter telomeres are targeted more efficiently by TERF2 inhibitor. In subsequent experiments they revealed that G4 stabilization by RHPS4 makes cells with longer telomeres (M14 cells) sensitive to TERF2 inhibition [136]. This indicates that G4 stabilization might induce telomere uncapping, which supports co-treatments that target short or unprotected telomeres (e.g. after TERF2 inhibitors) [136].

### Pancreatic cancer

Pancreatic cancer is associated with a poor prognosis. The five-year OS rate of diagnosed patients is only 9% [144]. Once surgery is incapable of removing the tumor, treatment options are very limited. In contrast to melanoma, pancreatic cancer is associated with a lower mutational burden [111] although 97% of pancreatic cancers have gene alterations [145].

*RAS* genes (*KRAS*, *NRAS and HRAS*) represent the most frequently mutated oncogenes in human cancer [146] including pancreatic cancer [147]. Generally, tumor cells harbor multiple genetic and epigenetic abnormalities. Nevertheless, in some cancers, tumor growth depends on one single oncogene and its continued activation. This is named oncogene addiction [148, 149]. For the initiation of pancreatic ductal adenocarcinoma (PDCA), the oncogenic *KRAS* mutation is indispensable [150]. In pancreatic cancer mutations in the oncogene *KRAS* are essential for tumorigenesis and impact directly multiple metabolic pathways of PDAC [150].

As *KRAS* is a driver of oncogene addiction, it is tempting to identify and characterize a therapeutic target to specifically down-regulate KRAS. The expression of all three *RAS* genes can be reduced by G4 formation [75, 151–153]. G4 stabilization by G4 ligands is a promising strategy to target the activity of the *KRAS* gene promotor [70]. There are three G4 motifs in the *KRAS* promoter which are located in the nuclease hypersensitive element upstream of the transcription start site [75, 154–157]. It has been reported that the transcription factors MAZ and HNRNPA1 as well as HMGB1 can upregulate *KRAS* expression by binding to a G4 structure within *KRAS* [158, 159]. G4 structure formation itself acts as a gene silencer for *KRAS* expression [75, 160]. These biochemical studies strengthen the hypothesis that G4 structures within *KRAS* represent a promising new drug target.

G4 ligands were also used in targeted approaches in pancreatic tumors analog to other cancer entities [72, 161]. These studies revealed that application of different G4 ligands resulted in reduced viability and growth inhibition of pancreatic cancer cells. In detail, the G4 ligand TMPyP4 induced tumor cell death when incubated with MIA Pa-Ca-2 pancreatic cancer cells while causing no effect in non-malignant cells [162]. Additional studies demonstrated that the G4 ligand MM41 led to reduced tumor cell growth in MIA Pa-Ca-2 xenografts [163]. Also, the G4 binding porphyrins Tetrakis and Octaacetyl inhibited proliferation of Ehrlich Ascites Carcinoma (EAC) solid tumors. The authors explained these growth changes of MIA Pa-Ca-2 and EAC tumors by the reduced expression of the *KRAS* and *BCL2* genes in cancer cells. Note, in these experiments the G4 ligands enhanced the expression of pro-apoptotic molecules such as BAX and TP53 and by this promoted apoptosis [164]. Similarly, the G4 ligand nitidine, or TINA-modified oligonucleotides (insertion of (R)-1-O-(4-(1-pyrenylethynyl)phenylmethyl]glycerol to increase stability) that mimic G4 structures of *KRAS* down-regulate KRAS protein expression and inhibit pancreatic cancer cell growth [155, 160]. One possible explanation for these observations is that *KRAS* expression is reduced due to the competition of G4 structure formation and MAZ binding [155, 165]. An alternative, but not exclusive hypothesis is that the G4 structures within the promoter are crucial for *KRAS* expression itself by inducing a positive feedback loop. KRAS stimulates the expression of ILK, which regulates HNRNPA1. HNRNPA1 binds and breaks down the G4 motif in the *KRAS* promotor and induces *KRAS* transcription [166, 167].

While most of the G4-related studies in the context of pancreatic cancer focus on changes of *KRAS* expression, one study demonstrated that G4 stabilization by BMSG-SH-3 in MIA Pa-Ca-2 xenograft tumors led to telomere shortening caused by reduced expression of TERT. In this study neither KRAS nor BCL2 expression changed [168].

Taken together, these studies revealed promising experimental evidence that G4 structure formation alters the expression of *RAS* genes and by this inhibits tumor growth. This can be explained by the blocking of DNA polymerases and/or altering the binding of proteins in these regions. It is not clear which additional G4 motifs are targeted by G4 ligands and if this induces genome instability, alters transcription or even translation of multiple other sites. It would be interesting to identify proteins that bind to G-rich non-G4 regions whose binding behaviour is altered upon G4 structure formation at oncogenes These proteins would be useful future drug targets to gain specificity of treatments with G4 ligands (Table 2).

**Table 2:** Overview of studies investigating the effect of G4 ligands on different pancreatic cancer cell lines

Ligand/G4 targeting	Cell line (all human)	Treatment duration (***in vitro***)	Growth Effect	Cellular Effects				Literature
				Telomere maintenance	Oncogene regulation	Genome instability	Not tested	
4,11-bis(2-aminoethylamino) anthra[2,3-*b*]furan-5,10-dione (2a),11-bis(2-aminoethylamino) anthra[2,3-*b*]thiophene-5,10-dione (2b)	PANC-1	72 h	IC_50_(metabolic activity): 0.26 μM (2a) and 0.9 μM (2b)		x			[169]
5ME	PANC-1	48 h	IC_50_: 80 μM				x	[122]
Alkyl-modified porphyrins	PANC-1	72 h	IC_50_(metabolic activity): ~15 nM		x			[142]
Azidothymidine (AZT)	MIA PaCa-2	4 and 7 days	IC_50_: >200 μM				x	[162]
BMSG-SH3	MIA PaCa-2	n.a.	50% decreased tumor growth of MIA-Pa-Ca2 xenografts No *in vitro* cyto-toxicity assay done	x				[168]
C14	PANC-1	n.a.	IC_50_: ~10 nM when irradiated with halogen light		x			[141]
C-2028	PANC-1, MIA PaCa-2, BXpC-3, AsPC-1, Capan-2	72 h	IC_50_ for all cell lines < 100 nm About 80% reduced Panc-1 xenograft growth *in vivo*		x			[170]
CM03	MIA PaCa-2, PANC-1	96 h	IC_50_: 7 nM (MIA), 18 nM (PANC-1), reduced tumor growth by ~ 73%			x		[171–173]
Copper(ii) l/d-valine-(1,10-phen) complexes (complex 1a, 1b)	BxPC3, AsPC1	72 h	IC_50_: ~2 μM for both complexes in both cell lines			x		[174]
CX-3543 (Quarfloxin)	MIA PaCa-2	n.a.	>50% reduced tumor growth of MIA PaCa-2 xenografts		x	x		[129]
CX-5461	Gemcitabine-resistant MIA PaCa-2 (GemMIA-R3) and normal MIA-PA-Ca2	96 h	GI_50_: 90.3 nM (GemMIA-R3), 88.7 nM (MIA-Pa-Ca2)				x	[175]
MM41	MIA PaCa-2, PANC-1	96 h	IC_50_: 11 nM (MIA), 3 nM (PANC-1), ~80% reduced growth of MIA PaCa-2 xenografts *in vivo*		x			[163, 171]
Naphthalene diimide ligands (compounds 3d, 3h)	MIA PaCa-2	96 h	IC_50_: 10 nM for both compounds	x		-		[45]
Naphthalene diimide isomer ligands (compounds 2-5)	MIA PaCa-2 PANC-1	96 h	IC_50_: 5-130 nM (MIA PaCa-2), 2 nM-1.5 μM (PANC-1)	-				[176]
Nitidine	AsPC-1, BxPC-3, MIA PaCa-2, PANC-1	72 h	IC_50_: 6.1 μM (AsPC-1), 5.2 μM (BxPC-3), 13.4 μM (MIA PaCa-2), 35.3 μM (PANC-1)		x			[160]
Octaacetyl	Panc-1_h_, MIA PaCa-2_h_	24-72 h	IC_50_: 65, 40, 36 μM for PANC-1 (24, 48, 72 h) and 62, 38, 33 μM for MIA PaCa-2 Reduced tumor growth *in vivo*		x			[164]
RHPS4	PAXF 736	15 days colony forming assay	IC_50_: 0.44 μM				x	[177]
SOP1812	MIA PaCa-2, PANC-1, Capan-1, BXPC-3	96 h	GI_50_: 1.3 nM (MIA PaCa-2), 5.9 nM (Capan-1) Significantly reduced MIA PaCa-2 xenograft growth *in vivo*		x			[173]
Telomestatin	MIA PaCa-2	48 h	IC_50_: 0.5 μM				x	[178]
Tetrakis	PANC-1, MIA PaCa-2	24-72 h	IC_50_: 60, 31, 25 μM (PANC-1) and 65, 36, 30 μM (MIA PaCa-2) for 24, 48, 72 h Reduced tumor growth *in vivo*		x			[164]
Tetrasubstituted naphthalene diimide ligands	PANC-1, MIA PaCa-2_,_ HPAC_,_ BxPc-3	96 h	IC_50_: 0.1-0.2 μM (PANC-1, MIA PaCa-2_,_ HPAC), 1.5 μM (BxPc-3)	x		x		[179]
TMPyP4	MIA PaCa-2, PANC-1	48 h, 4 and 7 days	IC_50_: 21.9 μM (MIA PaCa-2 , 4 days), 50 μM (PANC1, 7 days), 50 μM (MIA PaCa-2, 48 h), ~20 μM (PANC-1, 48 h). ~30% KRAS inhibition after 12 and 24 h		x			[122, 142, 154, 162, 178]

### G-quadruplexes and leukemia

Leukemia is caused by the abberant proliferation of blood cells. Different subtypes of leukemia exist according to the WHO classification depending on the affected cell type, the cell phenotype, cytogenetic and molecular genetic alterations. Acute myeloid leukemia (AML) is the most common acute leukemia in adults and is characterized by a relatively low number of mutations [112]. It is a clinically as well as genetically heterogeneous disease and is also popular as a leukemia model in G4 research. Over the past two decades a number of different G4 ligands (e.g. SYUIQ-5 [180], APTO-253 [181], TMPyP4 [182], telomestatin [62] and Tel03 [183]) were used to test their effect on leukemia cell growth. Most of these studies demonstrated that leukemia cell growth was inhibited after G4 structure stabilization by G4 ligands *in vitro* and *in vivo* [62, 181, 182]. Similar to previously discussed cancer entities, studies in leukemia revealed that after G4 stabilization the expression of oncogenes changed as well as the function of telomerase. G4 stabilization by telomestatin led to apoptosis and telomere shortening in leukemia cells from four AML patients [184]. Similarily, telomere shortening and senesence was observed with the G4 ligand SYUIQ-5 in K-562 and HL-69 leukemia cells, because of changes in the expression of telomerase (TERT) and TERF2 [180]. In addition to the relevance of G4 structure regulation at telomeres, several important leukemic oncogenes were inhibited after using G4 ligands (including *BCL2* [183], *MYC* [180, 181, 185], *MLLT1* and *AFDN* [186], *WT1* [182], *KIT* [181] and *KRAS* [185]) in human AML-derived cell lines.

As stated above, G4 structure formation might also challenge genome stability and exhibit tumor/leukemia-promoting properties. Indeed, Tauchi and collegues demonstrated that telomestatin activates the ATM-dependent DNA-damage checkpoint response [62]. *In silico* analysis revealed that 70% of the rearrenged genes in leukeamia contain a G4 motif [187]. This might indicate that G4 structures stimulate genome instability in these tumors. This was further supported by the finding that sites of rearrangement in TCF3, which promotes leukemia development, colocalize with regions that can form G4 structures [188]. In the future, it would be interesting to determine, if the G4 landscape within these mutagenic tumors changes and to address, which G4 structure newly forms and which are lost in these tumors. One hypothesis is that G4 structure formation contributes to the mutagenic nature of tumors. The determination of the location of G4 structures within these cancers together with deeper knowledge of the function and relevance of these structures will be beneficial to adjust G4 structure-driven treatments.

One of the most common recurring genetic events in AML are mutations in the C-terminal domain of *NPM1*, which occur in about 30-35% of all AML patients [189]. NPM1 is an abundant non-ribosomal nucleolar protein and essential for ribosome biogenesis. It was reported that NPM1 can bind to G4 structures located within the ribosomal DNA *in vitro* [190] and *in vivo* [191]. G4 binding by NPM1 is associated with its cellular localization. Experiments using the G4 ligand TMPyP4 in OCI-AML2 cells demonstrated that after G4 stabilization NPM1 translocates from the nucleolus to the nucleoplasm. The authors explain this effect by the competitive effect of TMPyP4 on G4 structure binding by NPM1 [191]. These findings together with the observation that the most common AML variant of NPM1 no longer binds to G4 structures in ribosomal DNA [191] led to the idea that the disruption of the G4 structure binding capability of NPM1 is linked to nucleolar localization in AML-associated protein variants [191].

As a side note we would like to point out that also G4 structure formation within RNAs could impact tumorigenesis and might be a useful target for cancer therapy. The translational control of oncoprotein expression is considered to be relevant in many solid cancers [192] and leukemias. EIF4A is an RNA helicase that promotes and sustains T-acute lymphoblastic leukemia and preferably targets mRNAs with G4 motifs in their 5’UTR [192]. Silvestrol, hippuristanol and pateamine A are natural compounds that target EIF4A. Inhibition of EIF4A with silvestrol showed anti-tumor efficacy *in vitro* and *in vivo* [192]. It was proposed that after EIF4A inhibition by silvestrol more G4 structures are formed within these transcripts leading to altered translation, which caused the slow growth of tumor cells (Table 3).

**Table 3:** Overview of studies investigating the effect of G4 ligands on different leukemia cell lines

Ligand/G4 targeting	Cell line (all human)	Treatment duration (***in vitro***)	Growth Effect	Cellular Effects				Literature
				Telomere maintenance	Oncogene regulation	Genome instability	Not tested	
1,8-dipyrazolcarbazole (DPC) derivatives, compound 7b	HL-60	48 h	IC_50_: 1.2 μM		x			[193]
7-substituted-5,6-dihydrobenzo[*c*] acridine derivatives, compound 2b	K-562	48 h	IC_50_: 9.2 μM		x			[194]
9-*N*-substituted berberine derivatives, compound 2j	HL-60	8 days	IC_50_: ~3 μM		x			[195, 196]
12-*N*-Methylated 5,6-dihydrobenzo[*c*] acridine derivatives, compound 21c	Ramos, CA46	96 h	IC_50_: ~5-10 μM		x			[197]
Actinomycin D	Ramos, CA46	24 h	IC_50_: ~25 nM (Ramos), ~10 nM (CA46)		x			[198]
Alkynylplatinum(II) terpyridine complexes (1-3)	K-562	48 h	IC_50_: 4-7.3 μM				x	[199]
APTO-253	AML: EOL-1, HEL92.1.7, MV4-11, KG-1, SKM-1, THP-1, NOMO-1, HL-60, MOLM-13 Leukemia/ Lymphoma: GRANTA-519, Jeko-1, Jurkat, SUDHL-6, Mino, Raji, Ramos	5 days	LC_50_: 0.14-1.75 μM (AML), 0.057-0.52 μM (non-AML)		x	x		[181]
AQ1	HMC1.2, α155	n.a.	n.a.		x	x		[200, 201]
B5	Ramos, CA45	48 h	IC_50_: 11.3 μM (Ramos), 21.8 μM (CA45)		x			[198, 202]
Ber8	HL-60	48 h	IC_50_: 1.7 μM	x	x	x		[196]
BRACO-19	CLL, AML	n.a.	IC_50_: 80 μM					[203]
C2	Ramos, CA45	48 h	IC_50_: 1.4 μM (Ramos), >100 μM (CA45)		x			[198, 202]
CORON	CCRF-CEM, HL-60(TB), K-562, MOLT-4, RPMI-8226, SR	n.a.	LC_50_: 0.85 μM (K-562), 1.4 μM (MOLT-4), 1.9 μM (HL-60(TB)), > 50 μM (all other)	x	x	x		[127]
CX-3543 (Quarfloxin)	A3, CCRF-CEM, D1-1, GDM-1, HL-60, I 9.2, J45-01, Jgamma-1, Jurkat, K-562, Kasumi-1, KG-1, Ku-812, MEG-01, MOLT-3, MOLT-4, MV-4-11, P116, Reh, RPMI-8226, RS4-11, SR, TF-1, THP-1	n.a.	IC_50_: ~1 μM	-		-		[129]
D2	Ramos, CA45	48 h	IC_50_: 11.7 μM (Ramos), 17.8 μM (CA45)		x			[198, 202]
Diquinolinyl- Pyridine Ligands (1a-c)	K-562, HL60	72 h	IC_50_: >50 μM (1a), 3 μM (1b), >50 μM (1c, all K-562) IC_50_: >50 μM (1a), 18 μM (1b), >50 μM (1c, all HL60)	x				[204]
Disubstituted quindoline derivatives, compound 74a	Raji CCRF-CEM U266B2	48 h	IC_50_: 4.7 μM (Raji), 18.1 μM (CCRF-CEM), 23.0 μM (U266B2) Xenograft of Raji cells >50% reduced tumor growth		x			[205]
DNR	K-562	48 h 96 h	IC_50_: 0.33 μM, 0.067 μM		x			[206]
EMICORON	CCRF-CEM, HL-60(TB), K-562, MOLT-4, RPMI-8226, SR	n.a.	LC_50_: 3.7 μM (HL-60(TB)), > 50 μM (all other)	x	x	x		[127]
GQC-05	KG-1a, CMK, TF-1	24 h	IC_50_: >1 μM		x	x		[207]
LZ-11	HL-60	48 h	IC_50_: 9.6 μM	x				[208]
MXR	K-562	48 h 96 h	IC_50_: 0.411 μM IC_50_: 0.105 μM		x			[206]
Pegaharmine D	HL-60	72 h	IC_50_: 3.81 μM				x	[209]
Pyrazine-based cyclometalated (C^N^pz^^C)Au(III) carbene complexes, compound 2 and 3	HL-60	72 h	IC_50_: 0.31 μM (2), 4.05 μM (3)				-	[210]
Pyridine(2,4- dihydroxybenz aldehyde dibenzyl semicarbazone) copper(II)	MOLT-4	24 h 72 h	IC_50_: 5 μM IC_50_: 1.5 μM		x			[185]
QPB compound **15e**	HL-60	48 h	IC_50_: 1.7 μM	x				[211]
Quinolino-benzo- [5, 6]-dihydro isoquindolium compounds 3a**,** 3f**,** 3g**,** 3j	HL-60	48 h	IC_50_: 2 μM (3j) - 12.6 μM (3g)		x			[212]
TMPyP4	K-562, OCI-AML2, OCI-AML3	48 and 96 h	IC_50_: 100-170 μM (K-562, 48 h), 60 μM (OCI-AML2, 96 h), 50 μM (OCI-AML3, 96 h)		x			[182, 191, 213, 214]
Telomestatin	Primary blast cells from AML patients OM9;22, K-562	10-30 days	IC_50_: 5 μM (10 days), 2 μM showed growth inhibition (15/30 days)	x		x		[62, 184, 215]
Substituted salicylaldehyde dibenzyl semicarbazones copper(II) complexes (7, 9, 7-py and 9-py)	MOLT-4	24 h	IC_50_: 3.1 μM (9-py), 18.1 μM (7-py), 5 μM (7), 8.03 μM (9)				x	[215]
Sysu12d	CA46, HL-60	48 h	IC_50_: 11.2 μM (CA46), 5 μM (HL-60)		x	x		[216]
SYUIQ-5	K-562, HL-60	72 h	IC_50_: 5 μM (K-562, HL-60) or 2.65 μM (K-562)	x	x	x		[180, 217]
SYUIQ-FM05	K-562	24 h	IC_50_: 10.83 nM		x			[218]

## Discussion

The research of the past decades links G4 structure formation and unfolding to defects and changes observed in different cancer entities. Current studies revealed that although most G4 ligands result in changes of cancer cell growth, they act via different mechanims and most likely at different targets. It is not clear, if the reduced growth effect on cancer cells after G4 ligand treatment is caused by multiple G4 structure-mediated changes, by a single G4 structure-driven event or by unspecific binding of the ligand. The problem in the current literature is to pinpoint the exact cause that drives growth changes and to monitor where the G4 ligand binds. Currently, there are several trials to establish G4 ligands that are specific to only one target [83, 219] and/or to develop novel photosensitizing G4 ligands that only cause toxic effects to G4 regions after irradiation [66]. These trials are very promising as it is expected that these approaches will reduce side effects. Additionally, the list of G4-interacting and -regulating proteins is increasing, which will not only give insights into G4 function in normal cells but will also provide ideas how to target/regulate specific G4 structures via the protein itself or protein-specific inhibotors. This would be particularly interesting for cancers like pancreatic cancer that for some cases are caused by a single upregulation of one oncogene (e.g. KRAS). In these cancers a G4 structure-based block of transcription is a very promising treatmen option. The current literature demonstrates that G4 stabilization often correlates with reduced cellular growth of cancers. It is not fully understood if the reduced growth is due to changes in telomere maintenance, transcriptional changes or genome stability. We would like to emphasize that co-treatment of G4 stabilization with drugs that either block DNA repair or induce additional genome instability is currently a very promising approach.

An interesting therapeutic strategy could be to combine immune checkpoint inhibitors with G4 structure-stabilizing ligands. It has been demonstrated that the success of immune checkpoint inhibition correlates with the mutational burden of a tumor [111, 112]. Hence, immunotherapeutic approaches might benefit from a combinational therapy with G4 ligands. It seems plausible that G4 ligands, which induce genome instability, might increase the immunogenecity of a tumor and sensitize it for checkpoint inhibition or other immunotherapeutic approaches.

## Conclusion

In this review we presented and discussed the relevance of G4 structure formation and stabilization as a therapeutical approach to treat cancer cells based on the current literature. As pointed out, different ligands have often a negative effect on cancer cell growth via different mechanisms. We discussed the hypothesis, if the mutagenic burden of the tumor positivly or negatively influences the outcome of G4 ligand treatment. We summarized the current research results that are linked to changes in G4 levels in melanoma, pancreatic cancer and leukemia cells. The conclusions are not very clear. We oberve that all three tumor entities show reduced cell growth upon treatment with different G4 ligands and, depending on the ligand, also changes in telomere maintenance, gene expression of oncogenes and increased genome instability. A direct comparision is difficult, because different studies were often done using different conditions. Some ligands were used in at least two entities. TMPyP4, EMICORON and CORON were tested in all three selected entities. Although similar effects were documented, the timing of treatment as well as the used concentration differs in these different studies. These differences indicate that lower concentrations of G4 ligands (e.g. EMICORON) are required to induce G4 structure-driven toxicity in cancer cells that have a high mutagenic burden (see Table 1, 2 and 3). A similar trend was observed for BRACO-19 (28 μM in melanoma and 80 μM for leukemia). Further experimental data demonstrated that a treatment with G4 ligands further enhances the cytotoxic effect in cells that have high levels of genome instability (e.g. after radiation therapy, after PARP inhibitor treatment or in BRCA1-deficient cells). However, in studies that used quarfloxin (CX-3543) we could not find this correlation. Future studies with similar culturing conditions and additional molecular studies are required to proof this hypothesis.

Lastly, we would like to disucss the potential impact that G4 structure formation might have on the mutagenic burden of the tumor and what the consequences are for tumor development and tumor relapse after treatment. In general, G4 structure formation, if not regulated efficiently (this includes formation and unfolding), can stimulate genome instability, which includes muations and deletions and complex gross chromosomal rearrangements [28, 30, 220]. A computational study investigated how the mutational burden correlates with G4 structure formation [221]. They revealed by comparing the location of potential G4 forming sites with cancer-associated breakpoints (using the COSMIC database) a significant overlap, in particular in those cancers that harbor a mutation in TP53. This is underlined by compuational studies in melanoma cells that link G4 regions with mutational hot spots [187]. A recent study identified a direct correlation of G4 structure formation with mutational changes in different breast cancer entities [222]. This supports the notion that G4 formation indeed stimulates and influences mutation rates in different cancers and may also contribute to subtype classifications [222]

## Data Availability

Not applicable

## Abbreviations

20A: 1-ethyl-N-(phenylmethyl)-4-(tetrahydro-2H-pyran-4-ylamino)-1H-pyrazolo[3,4-b]pyridine-5-carboxamide
AFDN: Afadin, Adherens Junction Formation Factor
AML: Acute myeloid leukemia
APTO-253: 2-(5-fluoro-2-methyl-1H-indol-3-yl)-1H-imidazo[4,5-f][1,10]phenanthroline
ATM: Ataxia Telangiectasia Mutated
ATRX: ATRX Chromatin Remodeler
BAX: BCL2 Associated X, Apoptosis Regulator
BCL2: BCL2 Apoptosis Regulator
BLM: Bloom Syndrome RecQ Like Helicase
BMSG-SH-3: 2,7-Bis-[5-(4-methyl-piperazin-1-yl)-pentyl]-4,9-bis-[3-(4-methyl-piperazin-1-yl)-propylamino]-benzo[lmn][3,8]phenanthroline-1,3,6,8-tetraone
BRACO19: N,N′-(9-(4-(Dimethylamino)phenylamino)acridine-3,6-diyl)bis(3-(pyrrolidin-1-yl)propanamide) hydrochloride
BRAF: B-Raf Proto-Oncogene, Serine/Threonine Kinase
BRCA1: Breast Cancer Type 1 Susceptibility Protein
BRCA2: Breast Cancer Type 2 Susceptibility Protein
BRIP1/FANCJ: BRCA1 Interacting Protein C-Terminal Helicase 1
C14: C14H28-alkyl derivative of PMPyP4
ChIP-seq: Chromatin Immunoprecipitation and sequencing
CX-3543: Quarfloxin, 15-fluoro-N-[2-[(2S)-1-methylpyrrolidin-2-yl]ethyl]-18-oxo-14-(3-pyrazin-2-ylpyrrolidin-1-yl)-12-oxa-1-azapentacyclo[11.7.1.02,11.04,9.017,21]henicosa-2,4,6,8,10,13(21),14,16,19-nonaene-19-carboxamide
CX-5461: 2-(4-methyl-1,4-diazepan-1-yl)-N-[(5-methylpyrazin-2-yl)methyl]-5-oxo-[1,3]benzothiazolo[3,2-a][1,8]naphthyridine-6-carboxamide
EAC: Ehrlich ascites carcinoma
EIF4A: Eukaryotic translation initiation factor 4A
G4: G-quadruplex
GI_20_/GI_50_: 20%/50% growth inhibition
GPI-15427: 10-((4-Methylpiperazin-1-yl)methyl)chromeno(4,3,2-de)phthalazin-3(2H)-one
h: Hours
HaCaT: Human adult low Calcium high Temperature Keratinocytes
HMGB1: High mobility group box 1
HNRNPA1: Heterogeneous nuclear ribonucleoprotein A1
HR: Homologous recombination
HRAS: HRas Proto-Oncogene, GTPase
IC_50_: Half maximal inhibitory concentration
ILK: Integrin Linked Kinase
IO: Immune-oncology
IR: Ionizing radiation
IZTZ-1: Benzothiazole-2-carboxaldehyde-derivate
KIT: KIT Proto-Oncogene, Receptor Tyrosine Kinase
KRAS: KRAS Proto-Oncogene, GTPase
LC_50_: Half lethal concentration
MAZ: MYC-associated zinc finger protein
MLLT1: MLLT1 Super Elongation Complex Subunit
MM41: 4,9-Bis((3-(4-methylpiperazin-1-yl)propyl)amino)-2,7-bis(3-morpholinopropyl)benzo[lmn][3,8]phenanthroline-1,3,6,8(2H,7H)-tetraone
MYC: MYC Proto-Oncogenes c-myc, l-myc, n-myc
n.a.: Not available
NHEJ: Non-homologous end joining
NPM1: Nucleophosmin 1
NRAS: NRAS Proto-Oncogene, GTPase
NU7441: 8-(4-Dibenzothienyl)-2-(4-morpholinyl)-4H-1-benzopyran-4-one
olaparib: 4-[[3-[4-(cyclopropanecarbonyl)piperazine-1-carbonyl]-4-fluorophenyl]methyl]-2H-phthalazin-1-one
OS: Overall survival
PARP1: Poly [ADP-ribose] polymerase 1
PDCA: Pancreatic ductal adenocarcinoma
PDS: Pyridostatin, 4-(2-Aminoethoxy)-N2,N6-bis[4-(2-aminoethoxy)-2-quinolinyl]-2,6-pyridinedicarboxamide
PDT: Photodynamic therapy
PIF1: Petite Integration Frequency 1
POT1: Protection of telomeres 1
Pou3FS/BRN2: Pou class 3 homeobox 2
PRKDC: Protein Kinase, DNA-Activated, Catalytic Subunit
Rad51: RAD51 Recombinase
RAS: NRAS Proto-Oncogene, GTPase
RHOJ: Ras homolog family member J
RHPS4: 3,11-Difluoro-6,8,13-trimethylquino[4,3,2-kl]acridinium methylsulfate
ROS: Reactive oxygen species
RSK: P90 ribosomal S6 kinase
SYUIQ-5: N-(10H-indolo[3,2-b]quinolin-11-yl)-N',N'-dimethylpropane-1,3-diamine
TCF3: Transcription Factor 3
Tel03: N,N¢-Bis-(2-(dimethylamino)ethyl)-3,4,9,10-perylenetetracarboxylic acid diimide
Telomestatin: (1R)-4,8-Dimethyl-3,7,11,15,19,23, 27-heptaoxa-31-thia-33,34,35, 36,37,38,39,40-octaazanonacyclo[28.2.1.12,5.16,9.110,13.114, 17.118,21.122,25.126,29]tetraconta-2(40),4,6(39),8,10(38), 12,14 (37),16,18(36),20,22(35), 24,26(34),28,30(33)-pentadecaene
TERF2: Telomeric repeat binding factor 2
TERT: Telomerase reverse transcriptase
TINA: Twisted intercalating nucleic acid
TMPipEOPP: 5,10,15,20-tetra-{4-[2-(1-methyl-1-piperidinyl)ethoxy]phenyl} porphyrin
TMPyP4: Meso-Tetra (N-methyl-4-pyridyl) porphine tetra tosylate
TP53: Tumor Protein P53
VEGF: Vascular Endothelial Growth Factor A
WHO: World Health Organization
WRN: Werner Syndrome RecQ Like Helicase
WT1: WT1 Transcription Factor
ZnP1: Zn(II) 5,10,15,20-tetrakis(N-carboxymethyl-4-pyridinium)porphyrin
